# Anthelmintic activity of silver-extract nanoparticles synthesized from the combination of silver nanoparticles and *M. charantia* fruit extract

**DOI:** 10.1186/s12906-016-1219-5

**Published:** 2016-07-26

**Authors:** Md. Mamun Or Rashid, Jannatul Ferdous, Sujan Banik, Md. Rabiul Islam, A.H.M. Mazbah Uddin, Fataha Nur Robel

**Affiliations:** 1 Department of Pharmacy, Noakhali Science and Technology University, Sonapur, Noakhali, 3814 Bangladesh; 2 Department of Pharmacy, International Islamic University Chittagong, Chandgaon R/A, Chittagong, Bangladesh; 3 Department of Applied Chemistry and Chemical Engineering, Noakhali Science and Technology University, Sonapur, Noakhali, 3814 Bangladesh

**Keywords:** Silver Nanoparticles (AgNPs), Ag-Extract nanoparticles, *M. charantia*, Anthelmintic activity

## Abstract

**Background:**

Present study has been conducted to know the anthelmintic activity of polyaniline coated silver nanoparticles (AgNPs) synthesized from *Momordica charantia* fruit extract.

**Methods:**

By reduction of AgNO_3_ in presence of NaBH_4_, silver nanoparticles were prepared. After mixing silver nanoparticles and extracts, coating was given on nanoparticles using polyaniline. Prepared nanoparticles were characterized by Visual, UV, FTIR spectroscopy, SEM techniques, and TEM analysis.

**Results:**

The FTIR results implied that AgNPs were successfully synthesized and capped with bio-compounds present in the extract. The result showed that death times of worm were 35.12 ± 0.5 and 59.3 ± 0.3 minutes for *M. charantia* extract and Ag-nanoparticles individually. But when these two combined together, paralysis and death time fall drastically which were only 6.16 ± 0.6 and 9.1 ± 0.4 minutes respectively. Albendazole tablet was used as standard, which made worms death in 3.66 ± 0.1 minutes.

**Conclusion:**

Ag-Extract NPs showed strong anthelmintic activity against worm. This study has paved the way for further research to design new anthelmintic drug from the combination of *M. charantia* and AgNPs.

**Electronic supplementary material:**

The online version of this article (doi:10.1186/s12906-016-1219-5) contains supplementary material, which is available to authorized users.

## Background

The application of nanotechnology is being important day by day. Various types of nanoparticles are produced by nanotechnology. Nanoparticles are gaining more and more attention due to their attracting properties [1]. Although physical and chemical methods are more popular for synthesis of nanoparticle, the use of toxic compounds minimizes their applications. Due to this problem, safe eco-friendly green methods have a vital role in synthesizing nanoparticles [2]. It is well known that silver nanoparticles with their extraordinary properties have diverse in vitro and in vivo biological applications. Silver nanoparticles exhibited significant anthelmintic activity when compared to standard [3].

*Momordica charantia* L. (bitter melon) is one of the most important species of the family Cucurbitaceae. Its fruit and other parts of the plant have medicinal value [4, 5]. The fruit is consumed as part of the diet but they are also reported to possess a wide range of pharmacological activities; for example, hypoglycemic, antidiabetic, antifungal, ability to inhibit p-glycoproteins, antihyperlipidemic and antioxidant effects have all been reported. The fruit has been used traditionally as anthelmintic, antiemetic, carminative, and purgative, as well as for the treatment of anemia, jaundice, malaria, cholera etc [6]. The in vitro and in vivo trial has shown that 3 % aqueous extract of *M. charantia* whole fruit possessed anthelmintic activity against *Ascaridia galli* [7]. *M. charantia* contains a number of potential pharmacologically active constituents such as charantine, goyaglycoside, mormodicoside, mormodicoside 3β, 25-dihydroxy-5β, 19-epoxycucurbita-6, (23E)-diene, momordicine-I, karavilagenin, karavilagenin C, karaviloside, karaviloside, kuguacin, kuguacin A, kuguacin B, kuguacin E, 3,7,23-trihydroxy-cucurbita-5,24-diene-19-al, 3,7,25-trihydroxy-cucurbita-5, 23-diene-19-al,3,7-dihydroxy-25-methoxycucurbita-5, 23-diene-19-al [6].

Helminthiasis or worm infections have continued to be the major health hazard to majority of people living in developing countries [8]. Helminthes control in domestic animals is widely based on the use of anthelmintic drugs. The high costs of drugs have awakened an interest in medicinal plants as an alternate source of anthelmintic drugs. In this study, we tried to evaluate the anthelmintic effect of nanoparticles prepared by the combination of Ag-Extract (*M. charantia).* This is the first reported study detailing the anthelmintic potential of green synthesized silver nanoparticles using *M. charantia* fruit extract. This will thus open a new door with pharmacological basis for the treatment of intestinal worm.

## Methods

### Collection of materials

Fresh young *M. charantia* were collected from the local market of Noakhali, Bangladesh. Then the plant’s material (unripe fruits) for the study was identified and authenticated by the National Harberium, Mirpur, Dhaka. The voucher specimen no. was (Accession No. DACB 42292). Silver Nitrate (MERCK, Germany), NaBH_4_ (LOBA CHEMIE) and all other reagents used in this study were analytical grades and collected from the laboratory of the Department of Pharmacy, Noakhali Science and Technology University.

### Experimental worms

Adult earthworms (8-10 cm) belonging to species of *Pheretima posthuma* were collected for this study as its anatomical and physiological resemblance with the intestinal roundworm parasite of human beings [9]. Because of easy availability, earthworms have been used widely for the initial evaluation of anthelmintic compounds in vitro [10]. Adult earthworms were collected from the wet soil of Noakhali district of Bangladesh. After collection, the samples were washed with saline water to remove soil and undesirable matter.

### Preparation of *Momordica charantia* extract

40 gm of *M. charantia* were collected and washed thoroughly with double distilled water, cut into fine pieces and then boiled at 100 °C in 200 mL of double distilled water for 30 minute. Then the aqueous extract obtained was by filtering through Whatman filter paper. The resultant clear extract was cooled and stored in refrigerator for analyzing anthelmintic activities.

### Preparation of silver nanoparticles

Ag nanoparticles were prepared according to Creighton, 1979 with slight modification [11]. At first, 100mL aqueous solution of 1.0 X 10–3 M silver nitrate was mixed thoroughly with a 300-mL aqueous solution of 2.0 X 10-3 M sodium borohydride. Triply distilled water was used for solution making and both solutions were allowed to chill (0 °C) before mixing. After mixing, Ag ions were reduced and formed nanoparticles in aqueous medium. Nanoparticles (Ag) containing solution was transparent and turned yellow color during mixing. The stirring was continuing for an hour until the color of the solution become yellow, which indicates the stabilization of Ag ion. At this point, Ag nanoparticles were stabilizing enough for long run without any stabilizing agent. As particle concentration of the solution was very low, it was concentrated 10 times using a rotary vacuum evaporator.

### Synthesis of Ag-Extracts nanoparticles

Silver nanoparticles were mixed by magnetic stirrer for half an hour with *M. charantia* aqueous extract which was prepared previously. This uncoated combined nanoparticles needed to coat.

### Coating of Ag-Extract nanoparticles by polyaniline

Coating on the AgNPs-Extract were done according to the method of K. Gopalakrishnan et al., 2012 with slight modification [1]. At first, 2.7 g aniline which was dissolved previously at 300 ml deionized water and 68ml of 6 % H_2_O_2_ were added slowly with the uncoated Ag-Extract NPs solution within30 minutes at room temperature. This addition was continuing for 23 hours as the coating substances finely coat the nanoparticles. Produced coated nanoparticles were filtered and dried at room temperature for concentrating and further analysis (anthelmintic activity).

### Characterization studies

The formations of silver nanoparticles were characterized by visual assessment as color changes of solution indicate the formation of nanoparticles. Reduction of pure silver ions were monitored by measuring UV-Vis spectra of the reaction mixture. UV-Vis spectra were measured using Hitachi, U-2800 spectrophotometer model UV- Visible double beam. For characterizing, absorption spectra of the samples were taken 200 to 700 nm. FTIR spectra were also performed and recorded with a Fourier Transform Infrared Spectrophotometer (IR Prestige-21, Shimadzu) in the range of 4000–500 cm^−1^ at a resolution of 4 cm^−1^. Size, shape and morphology of nanoparicles were determined by using Scanning Electron microscopy (2600SN Hitachi, Japan) from BCSIR, Bangladesh and Transmission Electron Microscopy (HITACHI H-700, Japan) from the Departmental laboratory of Applied Chemistry and Biochemistry, Kumamoto University, Japan.

### Preparation of test materials

At first, 5 ml of previous prepared *M. charantia* extract (40 gm/200 ml) was taken in adequate distilled water to make the concentration 50 mg/ml. Later, the concentration of silver nanoparticles and Ag-Extract nanoparticles were also adjusted to make the similar concentration (50 mg/ml) of *M. charantia* for comparing the efficacy against worms. Normal saline water was used as blank and Albandazole as standard in this experiment.

### Experimental design

The anthelmintic assay was carried out according to the method of Ajaiyeoba et al., 2001 with slight modification. The earthworms were subdivided into five groups each containing three worms. The first Group (I) served as control which receive saline water, whereas Group (V) received Albendazole (standard). Group (II), Group (III) and Group (IV) were treated with *M. Charantia* extract, AgNPs, AgNPs + Extract respectively. Observations were made for the time taken for paralysis and death. The time of paralysis was noted when there is no visual movement of worms except shaken vigorously. On the other hands, death time of the worms were recorded after confirming that the worms neither moved when shaken vigorously nor when dipped in warm water (50 °C).

### Statistical analysis

Data were analyzed using one way ANOVA tests (SPSS software, version-20) followed by Dennett’s t - tests on each group *P* < 0.05 were considered statistically significant (*).

## Results and discussion

### Visual assessment of AgNPs

Silver nanoparticles formation was confirmed by visual inspection. Color change during synthesis of silver nanoparticles was closely monitored. Initial brownish yellow color of mixtures turned into dark brown color within 20 minutes indicated the synthesis of silver nanoparticles. The change of color may be due to the excitation of surface plasmon resonance effect and reduction of silver nitrate [12].

### Evaluation of Ag-NPs by UV spectrum

**Fig. 1:**
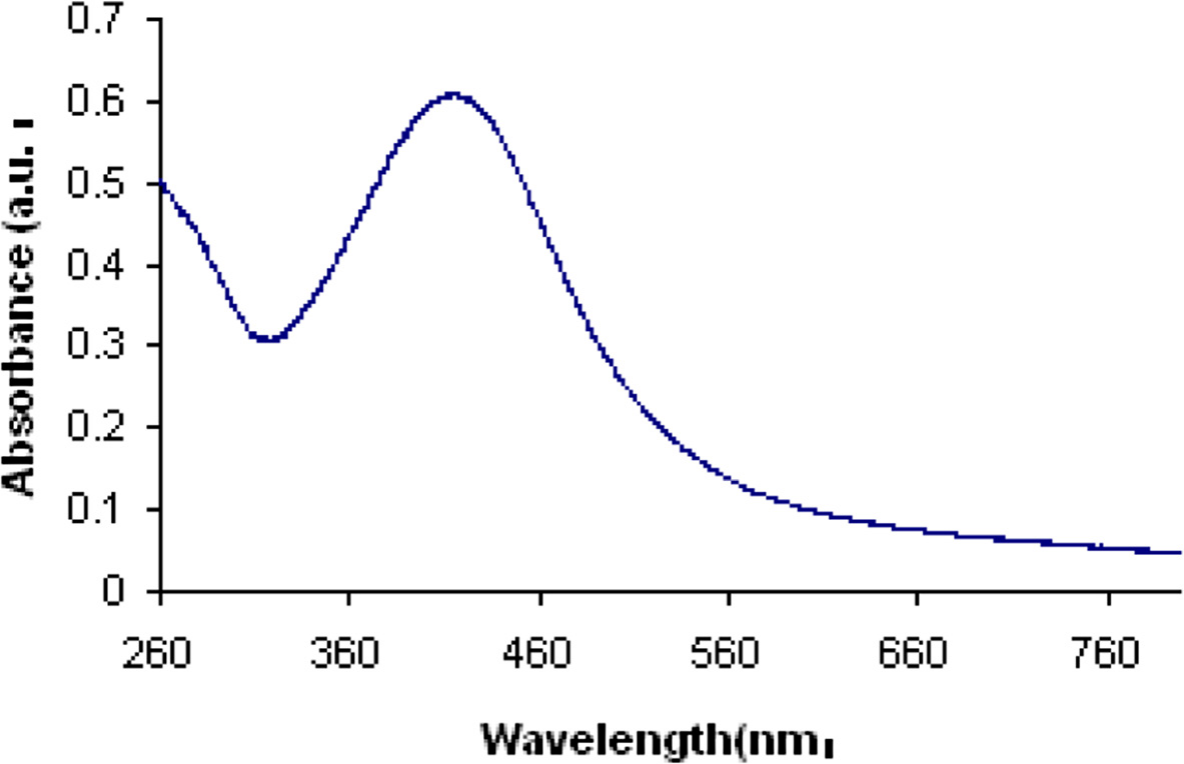
UV-Vis spectrum of AgNPs

An UV-Vis spectrophotometer (Hitachi, U-2800) was employed for the spectrometric analysis of prepared silver nanoparticles. The reduction of silver nanoparticles were measured in wavelengths ranging from 300 to 700 nm. It was seen that strong absorption peak was centering at approx. 400 nm (Fig. 1) which indicated the formation of AgNPs.

### Evaluation of Ag-Extract NPs through FTIR spectra

**Fig. 2:**
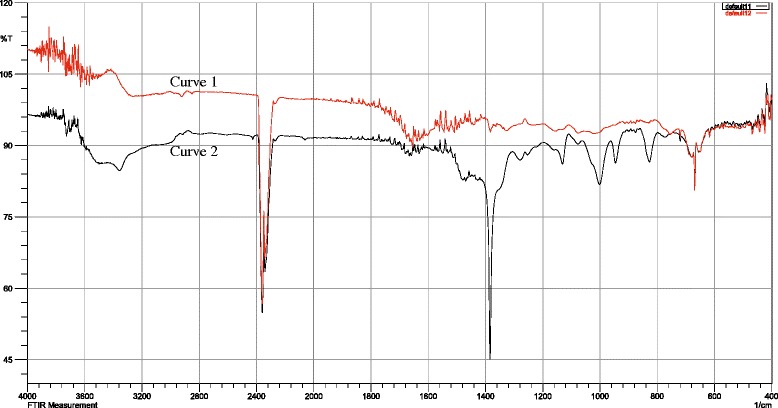
FTIR spectrum (**a**) *M. charantia* extract (Curve-1) (**b**) Ag-Extract NPs (Curve-2)

The FTIR spectra were used to identify the possible biomolecules (including functional group) responsible for the reduction of the Ag^+^ ions and capping of the *M. charantia* formed AgNPs. Figure 2, showed the FTIR spectra of *M. charantia* aqueous extract and bio-synthesized AgNPs. The possible functional groups of leaf extract involved in coating nanoparticle were identified by FTIR analysis. The intense absorption peaks at 3400.50 cm^-1^ (curve-1) and 3364.21 cm^-1^ (curve-2) correspond to N-H stretching of primary amine. The weak band observed at approx. 2900 cm^-1^ (curve-2) represents = C-H stretching. The weak band observed at 2926.01 cm^-1^ and 2880.79 cm^-1^ (both in curve-1 and curve-2) indicates the H-C-H asymmetric and symmetric stretching of alkanes. The band observed at 2341.58 cm^-1^ (curve-1) and 2368.94 cm^-1^ (curve-2), which are very nearer band, denotes the presence of hydrogen bonded OH stretching of carboxylic acids in leaf extract which may be a reducing/coating agent for silver nanoparticles.

The band at 1650.58 cm^-1^ (curve-1) and 1666.50 cm^-1^ (curve-2) represents N-H bending vibration of primary amine. The peak occurred at 1479.40 cm^-1^ (curve-2) for N-H bending vibration of second amine. C-N stretch occurred at 1400.02 cm. The band observed at 1332.96 cm^-1^ (curve-1) represent N = O stretching of nitro groups of leaf extract coated on nanoparticles. The arising of functional groups in FTIR spectrum indicates proper coating of leaf extract on silver nanoparticles. The bands at 1157.33 cm^-1^ (curve-1), which are nearly same in curve-b, 1130.29 cm^-1^ denoted C-H stretching vibration of ester. Beside, C-O stretch occurred at 100.08, 1072.47, 1026.17 cm^-1^ (curve-2) where first is stronger and broader than the second. The band at 915.12, 843.89, 829.43, 602.78, 668.36 cm^-1^ showed C-H bending vibration of alkynes. The band at 751.31 cm^-1^ and 719.45 cm^-1^ represented the ortho substituted and mono substituted aromatic stretching respectively. In addition, there is a band observered approximately 2400 cm^-1^ in both curves which indicates the presence of carbon dioxide in the air.

The FTIR results imply that the AgNPs were successfully synthesized and capped with bio-compounds present in the *M. charantia* extract by using a green method.

### Evaluation of Ag-Extract NPs by SEM and TEM

**Fig. 3:**
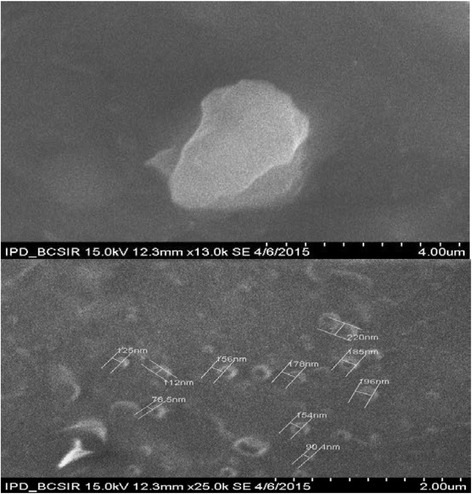
SEM image of Ag-Extract NPs

**Fig. 4:**
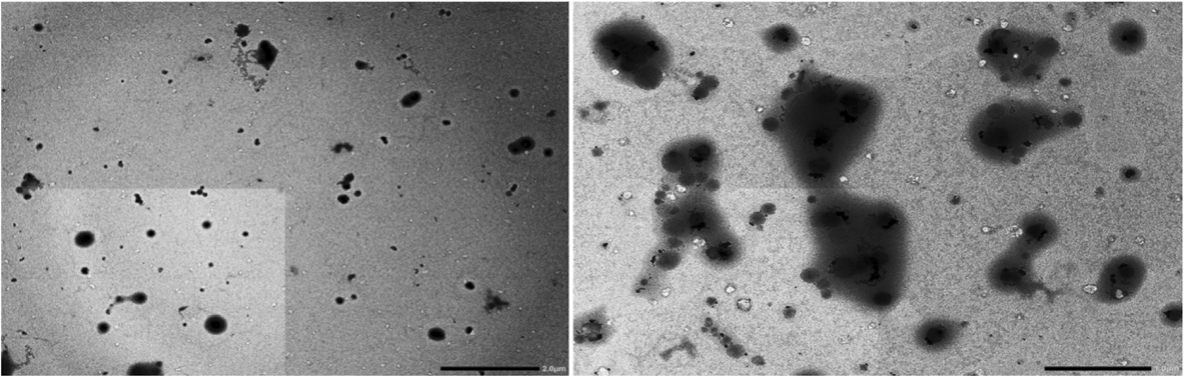
TEM image of Ag-Extract NPs

The SEM and TEM analyses showed the particle size between 78.5 to 100 nm in most cases; however few particles were larger than 100 nm (Fig. 3, Fig. 4). The nanoparticles were not in direct contact even within the aggregates, indicating stabilization of the nanoparticles by a capping agent. The larger silver particles may be due to the aggregation of the smaller ones, due to the SEM measurements. Most of the AgNPs-Extract nanoparticles were roughly circular in shape with smooth edges except some which were irregular in shape (Fig. 3).

### Assessment of anthelmintic activities

Anthelmintic activities of different groups were tested against the standard drug, Albendazole. Total 15 worms (three in each group) were taken in respective solutions. Then, the paralysis time and death time of the worms were recorded and the obtained results were shown in Table 1 (see details in Additional file 1: Table S1). This experiment was carried out upto 120 minutes and was compared with Albendazole (standard). Death time of worms by *M. charantia* extract and AgNPs was found 35.12 ± 0.5 minutes and 59 ± 0.3 minutes respectively. The mechanism of the inhibitory effects of Ag ions on microorganisms is partially known. It is reported that the positive charge on the silver ion is the reason for antimicrobial activity as it can attract the negatively charged cell membrane of microorganisms through the electrostatic interaction [13, 14]. On the other hand, *M. charantia* fruits consist glycosides, saponins, alkaloids, reducing sugars, resins, phenolic constituents, fixed oil and free acids [15]. These phytochemicals can attach with free proteins in the gastrointestinal tract or glycoprotein on the parasite cuticle and cause deaths. The combined anthelmintic activity of *M. charantia* and AgNPs was more effective than their individual effect, which was only 9.1 ± 0.4 minutes. Perhaps when extract was capped on Ag nanoparticles, these two components act as synergistic effect of each other. As a result strong wormicidal effect was found. The wormicidal activity of Ag-Extract nanoparticles against earthworms suggests that they are also effective against parasitic infections of humans. So it can be said that Silver nanoparticles combined with *M. charantia* extract gives strong anthelmintic effect.

**Table 1:** Anthelmintic activity of *M. charantia* extract, AgNPs and AgNPs + Extract

Test sample	Paralysis time (minutes)	Death time (minutes)
Normal saline	>120.00	>120.00
*M. charantia* extract (50 mg/ml)	16.76 ± 0.3*	35.12 ± 0.5*
AgNPs (50 mg/ml)	18.81 ± 0.2*	59.00 ± 0.3*
Ag-Extract NPs (50 mg/ml)	6.16 ± 0.6*	9.10 ± 0.4*
Albendazole (50 mg/ml)	2.50 ± 0.1	3.66 ± 0.1

Mean ± STD. Data were analyzed using one way ANOVA tests (n = 3). *p* < 0.05 were considered significant (^*^). Observation was done upto 120 minutes

## Conclusion

In this experiment, we tried to demonstrate a better method for developing anthelmintic agents in a simple, safe, cost effective and eco-friendly ways by using AgNPs and aqueous extract of *M. charantia.* The data generated from this experiment showed that Ag-Extract NPs possessed excellent anthelmintic activity against worm. This study has paved the way to design new anthelmintic drug from the combination of *M. charantia* and AgNPs. However, further investigation is required on human model to establish this finding for potential therapeutic effects.

## Abbreviations

SEM, Scanning Electron Microscopy; TEM, Transmission Electron Microscopy; FTIR, Fourier Transform Infrared Spectroscopy; UV, Ultraviolet; AgNPs, Silver Nanoparticles; Ag-Extract NPs, Silver Extract Nanoparticles

## Additional file

Additional file 1: Table S1. Anthemintic activity of Extract, AgNPs, and Ag-Extract-NPs. (XLSX 9 kb)
